# Defining Metal-Impurity Thresholds for Hydrogen Evolution in Sealed Vanadium Ion Batteries

**DOI:** 10.1021/acsomega.5c09587

**Published:** 2026-02-09

**Authors:** Dongyoung Lee, Bugi Kim, Eunhag Lee, Inwoo Cho, Dongheun Kim

**Affiliations:** Standard Energy, Daejeon 34014, Republic of Korea

## Abstract

The vanadium ion battery (VIB) has emerged as a next-generation aqueous energy storage system offering high safety, long cycle life, and scalability. Unlike redox flow batteries, the VIB employs a fully sealed architecture without external pumps, making suppression of the hydrogen evolution reaction (HER) a critical requirement. Here, we systematically investigate the role of metal ion impurities on HER in sealed VIBs. Representative impurities originating from vanadium mining, refining, and handling were introduced into the vanadium liquid electrode at concentrations up to 500 mg L^–1^ and internal pressure changes during cycling were monitored as a sensitive indicator of gas evolution. Distinct categories of impurity behavior were identified, ranging from strongly HER-promoting noble metals to species whose apparent inertness arises from limited solubility. From these results, impurity-specific threshold ranges were established, defining the onset concentrations at which HER leads to irreversible pressure build-up. These thresholds provide practical guidance for setting vanadium liquid electrode purity specifications and highlight the inherent differences between sealed VIBs and conventional redox flow batteries. Collectively, this work bridges fundamental understanding of impurity–HER interactions with industrial requirements for liquid electrode management, enabling long-term stability and safe operation of sealed VIB systems.

## Graphical abstract

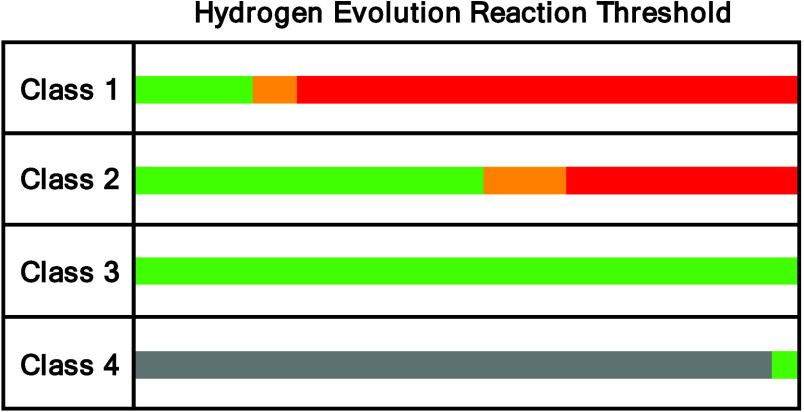

## 1 Introduction

The global transition to renewable energy has created an urgent demand for large-scale stationary energy storage systems that can stabilize power grids and ensure reliable electricity supply.^1,2^ Lithium ion batteries (LIBs)^3–5^ are the workhorse for mobile and increasingly stationary deployments, and other mature chemistries—including vanadium redox flow batteries (VRFBs),^6–11^ sodium ion batteries (SIBs),^12–15^ lead acid,^16–18^ and sodium sulfur (NaS) systems^19–21^ —serve distinct roles across the application spectrum. Within this diverse landscape, emerging grid-scale and high-power applications call for simultaneous ultrahigh power and ultralong cycle life with minimal balance-of-plant, motivating complementary sealed architectures that are optimized for both power delivery and durability.

The recently developed vanadium ion battery (VIB) is one such architecture: a sealed, pump-free configuration in which vanadium liquid electrode (electrolyte) operates with carbon-based solid electrode to deliver high round-trip efficiency, ultralong cycling, and ultrahigh-power capability suited to large-scale ESS and high-power applications. In our previous study, the VIB achieved round-trip energy efficiency exceeding 98% at 1 C-rate and stable operation beyond 12,000 cycles, demonstrating its durability and scalability.^22^ Unlike the conventional VRFB, which circulates electrolyte between external tanks and the electrochemical stack, the VIB confines all components within a compact sealed cell, eliminating pumps and reservoirs and thereby increasing volumetric power density and system simplicity. Because gas release and venting are more constrained than in VRFBs, mitigation of parasitic side reactions—particularly the hydrogen evolution reaction (HER)—becomes a key design requirement in sealed architectures. In this configuration, even minor hydrogen generation can gradually raise internal pressure, whereas in VRFBs, the gas can be relieved through external reservoirs or valves. Therefore, controlling the HER is essential for safe and stable operation.

Metallic impurities in vanadium liquid electrodes are recognized as key initiators of parasitic side reactions that deteriorate cell efficiency and lifetime. During the production and preparation of vanadium raw materials, such impurities can be introduced through upstream processes including mining, smelting, slag handling, and purification of vanadium pentoxide.^23–25^ Various extraction and leaching operations—such as roasting–leaching, reduction-assisted dissolution, and selective precipitation—have been reported to redistribute or incorporate metallic species such as Cu, Fe, Cr, and Mn into intermediate products.^26–32^ These metallurgical routes have therefore been identified as critical sources of impurity incorporation into vanadium liquid electrodes, and the overall effects of such impurities on cell performance and stability have been comprehensively reviewed in previous studies, even though the discussions were not specifically focused on hydrogen evolution.^33–37^ These impurities can catalyze the HER by lowering the overpotential at the negative electrode and facilitating hydrogen generation during electrochemical operation.^38–40^ To mitigate these effects, various purification approaches have been developed to reduce metallic impurities in vanadium resources and liquid electrodes, including roasting–leaching refinement, reductive or selective precipitation, photocatalytic chromium removal.^41–51^ However, most reported processes still rely on multistage thermal and chemical treatments that require substantial energy input and operational complexity, underscoring the necessity of establishing practical impurity-tolerance specifications instead of aiming for complete elimination.

In this study, we systematically investigate the influence of representative metal ion impurities on the HER in sealed VIBs. By introducing impurities at controlled concentrations and monitoring internal pressure fluctuations during charge–discharge cycling, impurity-specific threshold levels were identified. The resulting classification distinguishes severe HER promoters, moderate contributors, and ions with negligible impact or solubility. Collectively, this work defines impurity thresholds that connect materials-level phenomena to system-level performance requirements, providing both fundamental understanding of impurity-driven HER and industrially relevant guidelines for liquid-electrode quality control in sealed VIBs.

## 2 Experimental Section

### 2.1 Material Preparation

#### 2.1.1 Terminology

In this work, the term “vanadium liquid electrode” refers to the redox-active liquid phase—an aqueous sulfuric acid solution of vanadium ions spanning oxidation states from V­(II) to V­(V)—that stores and transports charge within the sealed cell. In conventional VRFBs, this solution also participates in redox charge storage but is referred to as the “electrolyte” because it circulates externally between tanks and the electrochemical stack. In the sealed VIB, the liquid phase is confined within the cell and directly functions as an active charge-storage medium, which we therefore refer to as the “liquid electrode.” The carbon felt serves only as an electronic conductor and reaction interface, distinct from the liquid phase that carries the redox-active species. This distinction clarifies that, although both systems rely on vanadium redox couples for charge storage, the VIB employs a structurally integrated liquid phase that functions as an electrode within the sealed architecture.

#### 2.1.2 Vanadium Liquid Electrode Preparation

A high-purity vanadium liquid electrode (Lotte Chemical Co., Ltd.) was employed, formulated as 2.1 M vanadium in 4.5 M sulfuric acid. This concentration corresponds to >23% increase in energy density compared with the conventional 1.7 M formulation widely adopted in VRFB research and industry. The 2.1 M composition was chosen not only because it enhances energy density, thereby improving cost competitiveness at the system level, but also because it can be stably manufactured by our supplier, ensuring practical scalability. At the same time, increasing vanadium molarity inherently raises the risk of hydrogen evolution, necessitating stricter impurity control—a central consideration of this study. The average vanadium oxidation state of the vanadium liquid electrode was +3.5, corresponding to an equal mixture of V^3^^+^ and V^4^^+^ species (V^3^^+^:V^4^^+^ = 1:1). This formulation provides a relevant baseline for impurity addition experiments, in which HER behavior was measured as a function of impurity concentration relative to the reference solution.

#### 2.1.3 Carbon Fiber Solid Electrode Preparation

Soft carbon fiber felt with a thickness of 5 mm (Gansu Haoshi Carbon Fiber Co., Ltd.) was employed as the electrode substrate for both the anode and cathode of the VIB. Prior to use, the carbon felt was subjected to a thermal activation treatment in a high-temperature furnace (Coretech Korea Co., Ltd.) at 550 °C for 2 h in ambient atmosphere. This process introduced oxygen-containing surface functionalities, enhancing the wettability and electrochemical activity of the electrode.^52^

#### 2.1.4 Separator Preparation

The polybenzimidazole (PBI) separator was selected for its ability to suppress vanadium ion crossover, which is critical for achieving high Coulombic efficiency in vanadium-based liquid systems. A 10% PBI solution was prepared by dissolving m-PBI (PBI Performance Products, Inc.) in dimethylacetamide (DMAc) under reflux at 160 °C for 24 h. To ensure homogeneity, residual particulates were removed by filtration through a 5 μm polyethylene (PE) filter. The PBI film was fabricated using a coater (DCN Co., Ltd.), where the solution was coated onto a 20 μm porous PE separator (Qingdao Lanketu Membrane Material Co., Ltd.). The porous PE layer served as a mechanical reinforcement and enhanced manufacturability, while the coated PBI layer provided selective proton transport and vanadium crossover suppression. After drying at 80 °C for 3 min, the reinforced PBI/PE composite separator was obtained and employed in the VIB cell.

#### 2.1.5 Current Collector Preparation

To improve manufacturability, the current collector was simplified to a single integrated component. Instead of assembling two separate parts—an aluminum current collector and a carbon current collector—a 100 μm-thick 1050-series aluminum foil was directly coated with a graphite film. This design reduced the part count from two to one, eliminating additional assembly steps while providing sufficient electrical conductivity and chemical stability. The integration not only enhanced process efficiency but also supports scalable and cost-effective production of VIB cells.

### 2.2 VIB Operation Mechanism and Cell Assembly

The VIB is designed as a compact sealed cell consisting of cathode and anode electrodes, a proton-conducting separator, and highly conductive current collectors. Each electrode is formed by a carbon-based conductive framework infiltrated with vanadium liquid electrode, where charge storage is achieved through redox transitions of vanadium ions rather than phase transformations, thereby minimizing irreversibility and ensuring long-term stability. In this configuration, the carbon fiber electrode functions as an electron pathway and reaction site, while the vanadium liquid electrode provides the active charge carriers, enabling rapid energy exchange. Proton migration across the separator maintains ionic balance, and the use of graphite-coated aluminum current collectors reduces resistive losses.

The cell assembly is shown in Figure 1. The VIB cell was assembled by inserting 126 mm × 126 mm carbon fiber electrodes into the cavities on both sides of a glass fiber reinforced plastic (GFRP) frame equipped with a separator and then covering each side with graphite-coated aluminum current collectors. A cross-shaped support was integrated inside the frame to resist separator deformation under compaction from the carbon fiber electrodes, thereby minimizing the volume change at both anode and cathode. Compaction pressure was applied using end plates, and additional fastening force was provided by PET tie bands to ensure structural integrity. After assembling the dry cell, a vanadium liquid electrode was vacuum-infiltrated through an injection port connected to the anode and cathode via two corresponding connecting channels. A total of 114.7 mL of liquid electrode, corresponding to 97% of the internal cell volume, was injected. The volume was intentionally kept 3% below the full capacity to accommodate physical expansion during changes in vanadium oxidation states. Finally, the injection port was sealed, completing the closed-cell architecture.

**Figure 1:**
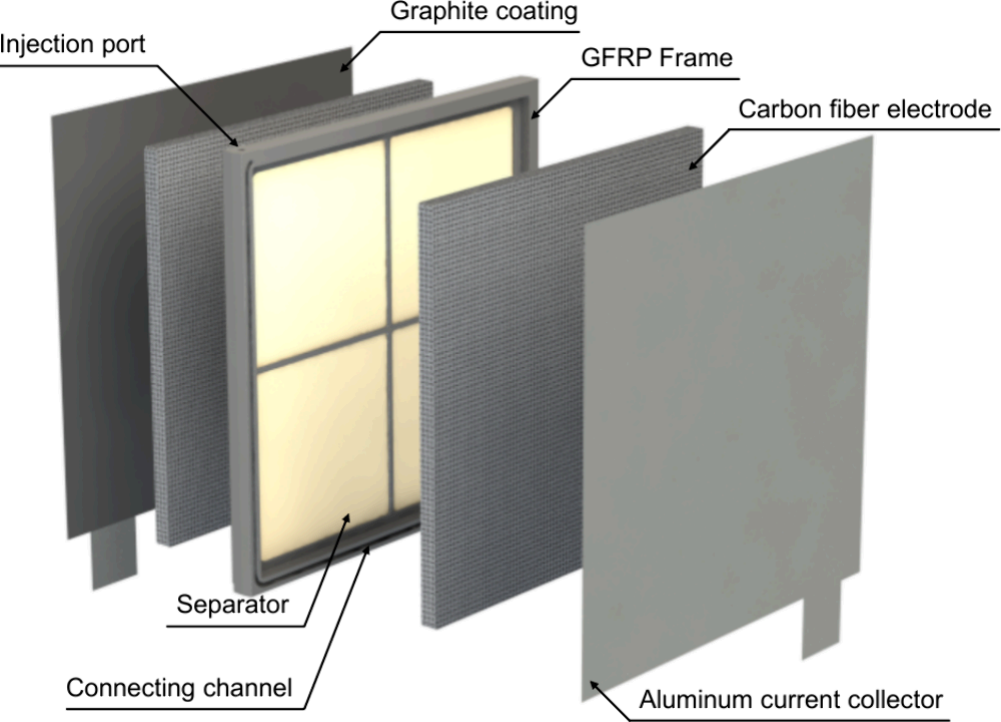
Schematic exploded view of the VIB cell assembly.

### 2.3 Addition of Impurity in Vanadium Liquid Electrode

To simulate impurity ingress, selected metal reagents were introduced into the vanadium liquid electrode at concentrations up to 500 mg L^–1^. Experiments were conducted at 3–4 different concentration levels to establish the impurity threshold range. In practical vanadium liquid electrodes, impurity concentrations are typically observed at trace levels (≪500 mg L^–1^). Accordingly, concentrations above 500 mg L^–1^ cannot be realistically regarded as impurities, but rather as deliberate additives, and were therefore excluded from this study. The impurity elements were selected based on their known presence in vanadium mining and refining processes, as well as prior reports identifying contaminants that promote the HER. Since certain elements were only partially soluble in the vanadium liquid electrode, any precipitates were removed by filtration through a PE filter and only the supernatant was collected. The dissolved fraction was then quantified using ICP-OES (Agilent 5800, Agilent Technologies).

In total, 30 impurity ions were investigated, including alkali metals (Li^+^, Na^+^, K^+^, Cs^+^), transition metals (Fe^2^^+^, Co^2^^+^, Ni^2^^+^, Cu^2^^+^, Mo^6^^+^, W^6^^+^, Cr^3^^+^, Mn^2^^+^), precious metals (Pt^4^^+^, Au^3^^+^, Ag^+^, Rh^3^^+^, Ir^3^^+^/Ir^4^^+^, Ru^3^^+^/Ru^4^^+^, Os^4^^+^, Pd^2^^+^), metalloids (Sb^3^^+^, Se^4^^+^, Te^4^^+^, Si^4^^+^), and post-transition metals (Bi^3^^+^, Pb^2^^+^, Al^3^^+^, In^3^^+^, Zn^2^^+^, Cd^2^^+^). The corresponding reagents and CAS numbers are summarized in Table S1.

### 2.4 Cell Testing and Hydrogen Evolution Measurement

The completed cell was equipped with a pressure transducer (PDK Co., Ltd., measurable range −100 to 100 kPa) to sensitively capture gas evolution while minimizing void volume in the overall design (Figure 2). The additional volume introduced by the transducer and connector was approximately 0.1 mL (<0.1% of the total internal volume) and thus had negligible influence on pressure changes.

**Figure 2:**
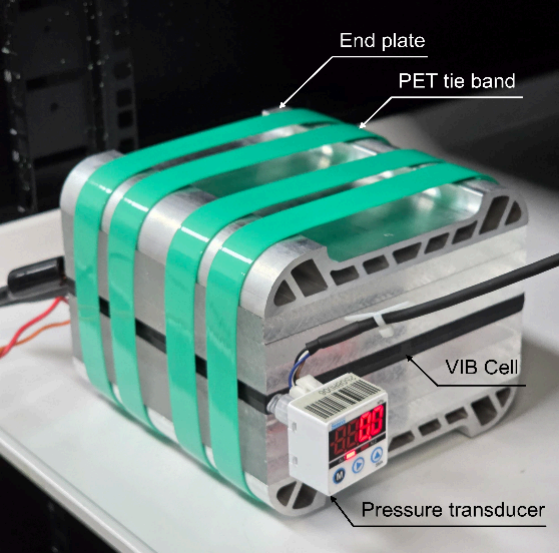
In situ pressure monitoring setup for hydrogen evolution measurement. All photographs were taken by the authors.

Galvanostatic charge–discharge cycling tests were performed using battery testers (Neware Technology Co., Ltd.). Each cycle consisted of a constant current (CC) charge at 2.2 A up to 1.6 V, followed by a constant voltage (CV) step until the charging energy reached 3.2 Wh. After a 100 s rest period to measure the open-circuit voltage (OCV), the cell was discharged at a CC of 2.2 A down to 1.12 V, followed again by a 100 s rest to record the OCV (∼1.2 V). The applied current density was 12.8 mA g^–1^, calculated based on the combined mass of both the solid (9.6 g) and liquid (162.6 g) electrodes, while the energy density was standardized to 18.6 Wh kg^–1^ (19.7 Wh kg^–1^ and 27.9 Wh L^–1^ when normalized solely to the liquid electrode). All experiments were conducted at 25 ± 1 °C.

Internal pressure was continuously monitored during cycling, and representative peak values were used to assess hydrogen evolution related trends. The impurity threshold level was conservatively defined as the concentration at which the internal pressure exceeded 50 kPa during cycling. This threshold was determined from structural considerations of the sealed VIB cell: the cell was vacuum filled and designed to withstand a negative pressure of −100 kPa, while under internal pressurization it can tolerate up to ∼100 kPa, beyond which minor leakage occurs by design to prevent excessive accumulation. By applying a safety factor of 2 to this structural limit, the impurity threshold for hydrogen evolution was conservatively set at 50 kPa.

## 3 Results and Discussion

### 3.1 Electrochemical Performance and Internal Pressure Fluctuations

During charge–discharge cycling, the density of the vanadium liquid electrode changes with the state of charge (SOC), which corresponds to changes in the vanadium oxidation state. Figure 3b shows the change of density measured by density meter (DMA 4100, Anton Paar) at 25 °C. At the cathode, V^4^^+^→ V^5^^+^ raises the density by ∼0.7%, while at the anode, V^3^^+^→ V^2^^+^ lowers it by ∼1.7%; taken together, the overall liquid electrode density decreases by ∼1%, i.e., the liquid electrode volume increases by ∼1% during charging. Because the VIB is sealed with fixed internal volume and limited headspace, this net volume increase cannot be freely accommodated and therefore manifests as a reversible pressure rise on charge and a corresponding decrease on discharge.

**Figure 3:**
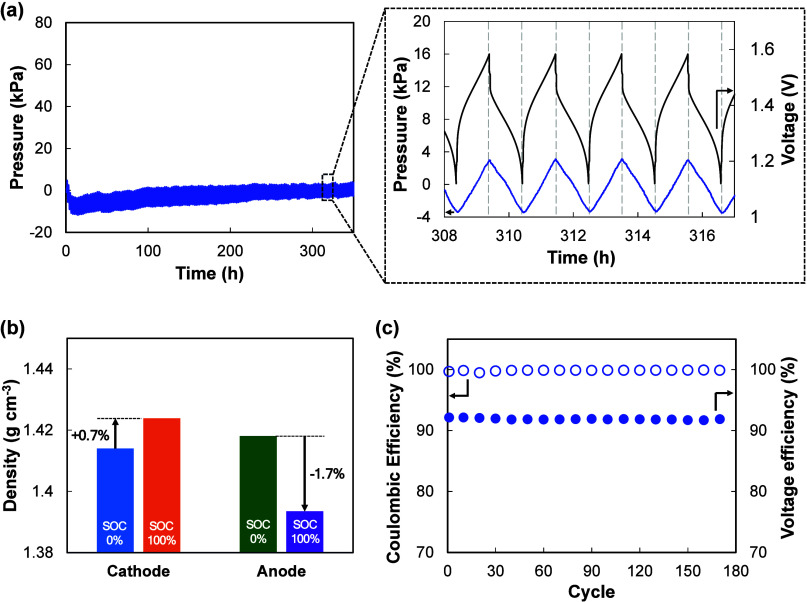
Electrochemical characteristics of a sealed VIB with the reference vanadium liquid electrode. (a) Stable cycling showing reversible pressure oscillations without accumulation, accompanied by representative charge–discharge profiles, (b) density change of the vanadium liquid electrode as a function of SOC, illustrating volume expansion during charging, and (c) electrochemical performance metrics, including Coulombic and voltage efficiencies.

The corresponding pressure response is illustrated in Figure 3a, where the internal pressure increases during charging and decreases again during discharging, reflecting the reversible density fluctuations. Over multiple cycles, the pressure oscillates between charge and discharge without accumulation, consistently returning to its baseline level. Notably, although the initial pressure was set to 0 kPa, a transient negative pressure was observed in the early cycles, followed by gradual recovery to near-zero. This phenomenon can be attributed to residual oxygen within the headspace: since the cell was not completely filled to allow for expansion, a small void space remained, and approximately 20% of the trapped air was oxygen. During early cycling, V^2^^+^ ions consumed oxygen, resulting in a temporary negative pressure. In addition, minor discrepancies in the injected volumes of vanadium liquid electrode supplied to the anode and cathode may contribute to small variations in the initial pressure.

The critical observation, however, is whether the internal pressure exhibits a continuous upward trend (indicative of gas accumulation and thus a safety risk) or remains reversible within a narrow range. In our reference cells containing only the pristine vanadium liquid electrode, no net pressure build-up was observed. Pressure fluctuations remained within approximately −20 to 20 kPa, without progressive increase during cycling. Based on our experimental experience, when no upward trend is observed within approximately 150 cycles (≈300 h of operation), the internal pressure tends to remain stable during extended cycling under identical conditions.

Two routine background factors account for the small excursions: (i) early cycle consumption of residual oxygen in the headspace, which can transiently drive the pressure down to ∼ −20 kPa; and (ii) ambient temperature variation of ± 1 °C, typically giving ∼2 to 4 kPa changes (occasionally up to ∼6 kPa). These minor effects do not compromise electrochemical performance—Coulombic and voltage efficiencies were unchanged (Figure 3c). The reference vanadium liquid electrode contained only trace level impurities by ICP-OES (Table S2), indicating that the stable pressure profile originates from intrinsic vanadium redox reactions with reversible volume change rather than impurity-driven effects.

### 3.2 Impurity Classifications and Impact

Based on their characteristic pressure responses, the impurity ions were categorized into four classes.

- Class 1 includes the most critical species, which triggered a steep internal pressure rise even at trace levels of only tens to hundreds of μg L^–1^, indicating the highest risk for hydrogen evolution.
- Class 2 consists of ions that induced rapid and significant pressure build-up at higher concentrations ranging from several to hundreds of mg L^–1^, representing the next tier of concern.
- Class 3 encompasses ions that did not induce hazardous pressure accumulation even when added up to 500 mg L^–1^, suggesting relatively benign behavior under the tested conditions.
- Class 4 covers ions that similarly showed no critical pressure increase, but whose solubility was intrinsically limited, leading to precipitation before reaching the 500 mg L^–1^ threshold.

#### 3.2.1 Class 1 – Pt, Rh, Pd

Noble metal ions such as Pt, Rh, and Pd exhibited the strongest catalytic activity toward the HER. Among them, Pt is presented here as the representative example, while the corresponding results for Rh and Pd are provided in the Supporting Information (Figure S1).

As shown in Figure 4, Pt ions induced rapid internal pressure accumulation even at trace concentrations. At 10 μg L^–1^ no discernible increase was observed, but at 25 μg L^–1^ a steep and irreversible rise in pressure occurred, clearly exceeding the 50 kPa threshold within early cycles. At 50 μg L^–1^ the internal pressure surpassed 80 kPa, eventually causing leakage and forcing termination of cycling only at the second cycle.

**Figure 4:**
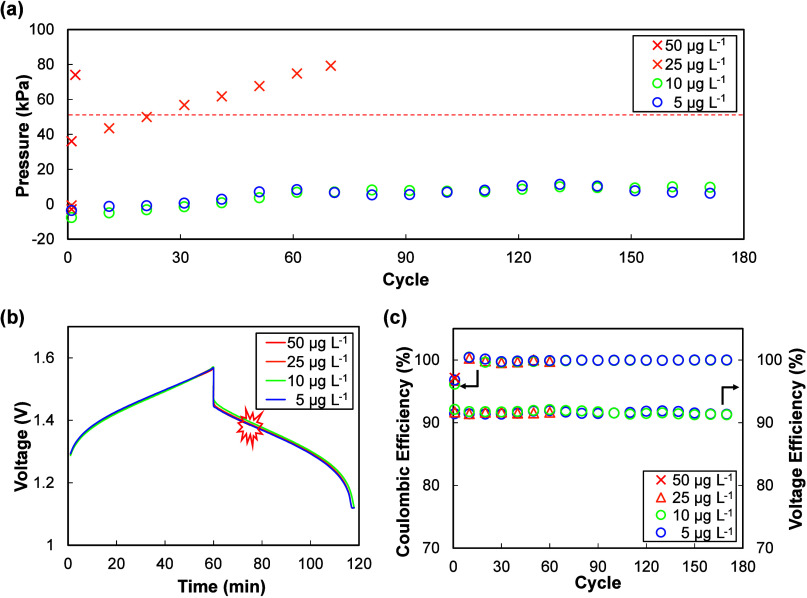
Class 1 (Pt) impurity effect. (a) Internal pressure evolution at different impurity concentrations, (b) representative charge–discharge curves, and (c) Coulombic and voltage efficiencies showing negligible changes despite pronounced hydrogen evolution.

Interestingly, Coulombic and voltage efficiencies remained essentially unchanged across all concentrations, even in cases with pronounced hydrogen evolution. This indicates that HER triggered by Class 1 impurities may not be readily detected from conventional electrochemical performance metrics. Instead, the pressure measurements provided a far more sensitive probe, directly capturing the onset and severity of gas generation that would otherwise remain hidden in charge–discharge curves.

#### 3.2.2 Class 2 – Ru, Cu, Os, Te, Ir, Ag, Au, Bi, Sb

A second group of impurities triggered hydrogen evolution at higher concentrations compared to Class 1, typically in the several to hundreds of mg L^–1^ range. Cu is presented here as the representative element (Figure 5), while the results for the other Class 2 ions are summarized in the Supporting Information (Figure S2).

**Figure 5:**
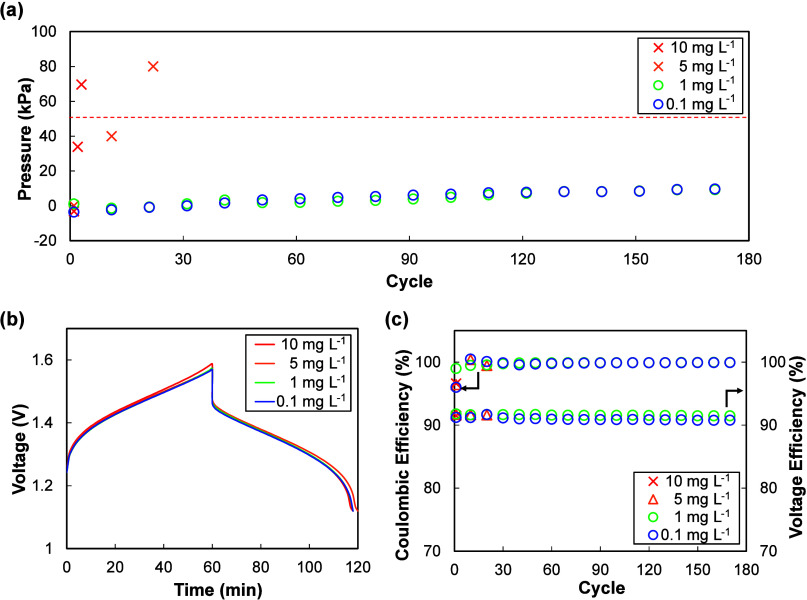
Class 2 (Cu) impurity effect. (a) Internal pressure evolution at different impurity concentrations, (b) representative charge–discharge curves, and (c) Coulombic and voltage efficiencies showing negligible changes despite pronounced hydrogen evolution.

As shown in Figure 5a, the internal pressure remained stable below 1 mg L^–1^, but began to rise rapidly above 5 mg L^–1^, and eventually exceeded the 50 kPa threshold. This behavior suggests that while these ions are less catalytically active for HER than Class 1 metal ions, they can still facilitate hydrogen generation once present above certain concentrations.

From a manufacturing standpoint, Class 2 ions are realistic contaminants in the vanadium value chain—arising from ore processing, hydrometallurgical refining, solvent extraction, or materials handling—and ppm-level residues are commonly reported even after purification. In particular, Cu (representative in Figure 5) is frequently noted in various processes, making their routine monitoring and specification important at the mg L^–1^ levels identified by our thresholds. Therefore, Class 2 impurities represent a moderate but realistic risk: while trace levels are benign, sustained accumulation to tens of mg L^–1^ could compromise long-term cell stability.

#### 3.2.3 Class 3 – K, Zn, Al, Na, Co, Cr, Li, Fe, Ni, In, Cd, Mn

A third group of impurities showed no significant influence on pressure or electrochemical performance, even at the highest tested concentration of 500 mg L^–1^. Representative data for Fe are shown in Figure 6, while the remaining Class 3 ions are summarized in the Supporting Information (Figure S3).

**Figure 6:**
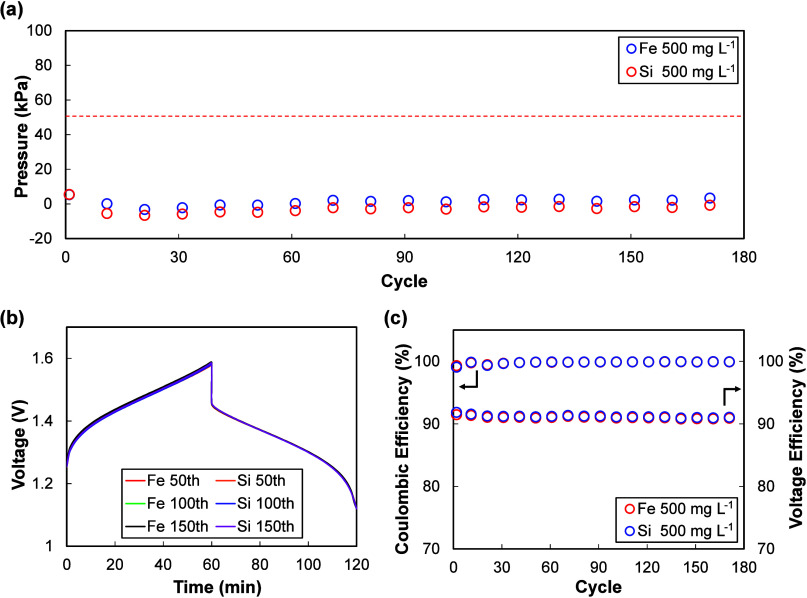
Class 3 (Fe) and Class 4 (Si) impurity effect. (a) Internal pressure evolution at impurity input concentration of 500 mg L^–1^, (b) representative charge–discharge curves, and (c) Coulombic and voltage efficiencies showing negligible changes.

As illustrated in Figure 6a, internal pressure fluctuations remained fully reversible and within the −20 to 20 kPa window defined by the reference cells, with no cumulative build-up. This indicates that these ions neither catalyze HER nor significantly interfere with the vanadium redox couples. From a mechanistic standpoint, species such as Fe, Mn, and Cr have intrinsically low HER activity and are unlikely to alter the electrode kinetics at the concentrations tested.

Importantly, Coulombic and voltage efficiencies (Figure 6c) were indistinguishable from those of the reference cell and showed no change of charge–discharge profile during cycling. This suggests that Class 3 impurities can be tolerated up to several hundred mg L^–1^ without posing measurable risks to either the electrochemical performance or the structural integrity of sealed VIBs. From a practical standpoint, this class defines the “pressure tolerance window” for impurities that may be present at residual levels in vanadium liquid electrodes without requiring strict purification.

#### 3.2.4 Class 4 – Mo, W, Pb, Si, Cs, Se

Finally, Class 4 impurities exhibited negligible effects not only because of intrinsically benign behavior but also because their effective concentrations were constrained by low solubility in the vanadium liquid electrode. Within this class, some species dissolved partially when nominally introduced at 500 mg L^–1^, whereas Si remained almost entirely undissolved. In practice, the undissolved precipitates were removed by filtration, and subsequent electrochemical testing was conducted using only the supernatant solution. The actual dissolved concentrations were quantified by ICP-OES analysis, and the tested values are indicated by the black diamond markers in Figure 7. It should be noted that this solubility-limited behavior differs from the electrochemical reduction that may occur for noble metal ions in Class 1, which may be reduced to metallic clusters on carbon surfaces because of their high redox potential.

**Figure 7:**
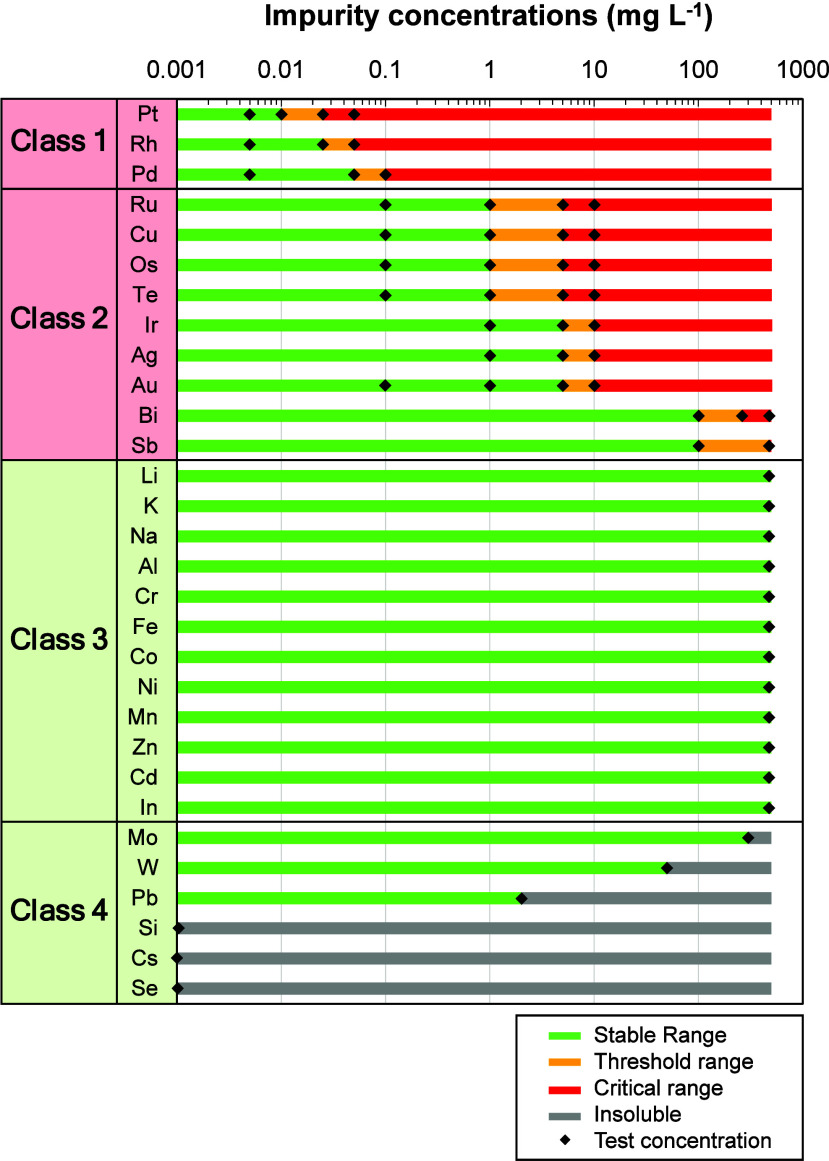
Impurity thresholds and classification of hydrogen evolution risk.

Representative data for Si are presented in Figure 6. No irreversible pressure build-up was observed, and Coulombic and voltage efficiencies were unaffected. This distinguishes Class 4 from Class 3: the negligible impact arises not only from the absence of catalytic activity but also from their limited solubility—rendering the electrochemical response essentially indistinguishable from the reference cell. Although Class 4 impurities do not directly trigger hydrogen evolution, their insoluble residues can interfere with liquid electrode handling, for example, by affecting dispensing processes if not removed. Fortunately, these precipitates can be effectively eliminated through simple filtration, which minimizes immediate risk. Nevertheless, in scaled-up systems such as mass production lines, accumulated solids may still pose challenges such as sludge formation, electrode fouling, or flow channel blockage. Therefore, while electrochemical performance remains unaffected in the short term, Class 4 impurities highlight a distinct category of risk associated with solubility and process management.

### 3.3 Summary of Impurity Threshold Level

The classification and thresholds of all investigated impurities are summarized in Figure 7, providing practical guidelines for defining impurity specifications in commercial vanadium liquid electrode production.

- **Green (Stable range)** – impurity concentrations at which no significant pressure rise was detected, indicating a tolerable level without hydrogen evolution.
- **Yellow (Threshold range)** – intermediate concentrations where the onset of hydrogen evolution was observed with concentration-dependent variability, representing the critical transition window.
- **Red (Critical range)** – concentrations at which impurities consistently induced pronounced and irreversible internal pressure rise due to hydrogen evolution.
- **Gray (Insoluble)** – impurities that remained practically undissolved even when introduced at 500 mg L^–1^, resulting in limited solubility under the investigated conditions.

The results obtained in this study can differ from those reported for conventional VRFBs because the operating architectures are fundamentally different. In VRFBs, the electrolyte is continuously circulated between a relatively small electrochemical stack and large external tanks; over long residence times the recirculating loop repeatedly exposes trace metallic species to large carbon surface area, so that even ppm-level residues can gradually adsorb, co-deposit, or nucleate precipitates on electrodes and flow components, leading to performance drift during extended operation. In contrast, the vanadium ion battery (VIB) operates in a sealed configuration with a fixed volume of vanadium liquid electrode without macroscopic forced convection. As a result, impurities are neither redistributed by bulk flow nor continually reintroduced to fresh electrode surface, and their effects at a given nominal concentration can be less pronounced than in VRFBs. This structural distinction confers a degree of inherent tolerance to many metallic impurities in VIBs, while underscoring that the thresholds reported here are specific to the sealed architecture and should not be directly extrapolated to flow battery conditions.

### 3.4 Limitations and Outlook

This study establishes impurity-specific thresholds for hydrogen evolution in sealed vanadium ion batteries using a 2.1 M V/4.5 M H_2_SO_4_ liquid electrode at 25 ± 1 °C, with single-impurity dosing and in situ pressure as the primary readout. The reported thresholds therefore reflect the tested architecture and operating conditions; variations in liquid electrode chemistry (acid composition and concentration), electrode microstructure, temperature window, and cycling protocol may shift the values. Anionic impurities and complexants were not mapped here and warrant dedicated study, as do mixed impurity cross effects (synergy/antagonism) that may emerge under realistic contamination profiles. Furthermore, as practical systems move toward even higher vanadium molarity to further boost energy density and cost competitiveness, impurity management will become increasingly critical, underscoring the need to extend such threshold mapping to higher-molarity regimes.

## 4 Conclusions

We quantified how representative metal ion impurities govern hydrogen evolution in sealed vanadium ion batteries (VIBs) and translated those findings into actionable threshold ranges. By coupling controlled impurity addition with in situ pressure monitoring, we identified the onset concentrations at which internal pressure irreversibly increases (≥threshold) and organized the impurities into four classes: (i) Class 1 that trigger HER at subppm levels; (ii) Class 2 exhibiting mg L^–1^ level thresholds; (iii) Class 3 that remain stable up to 500 mg L^–1^; and (iv) Class 4 whose apparent inertness stems from solubility-limited effective concentrations. The consolidated risk map—stable/threshold/critical/insoluble—provides a direct basis for liquid electrode quality control specifications in sealed architectures. Practically, it prioritizes tight control for Class 1, monitoring for Class 2 (relevant to mining/refining value chains), tolerance windows for Class 3, and filtration/process management for Class 4 (which may pose handling/maintenance risks despite electrochemical benignity). The framework clarifies why impurity behavior in VIBs can differ from VRFBs, where continuous circulation and large reservoirs repeatedly expose trace metals to fresh surfaces, whereas sealed VIBs lack such flow-driven redistribution. Overall, this work links impurity–HER interactions to industrial requirements for safe, long-life operation of sealed VIB systems.

## Supplementary Material



## Data Availability

The data supporting the findings of this study are available within the article and the Supporting Information. Additional underlying data are not publicly available due to ongoing intellectual property considerations but may be made available from the corresponding author upon reasonable request.

## References

[1] Kebede A. A., Kalogiannis T., Van Mierlo J., Berecibar M. (2022). A comprehensive
review of stationary energy storage devices for large scale renewable
energy sources grid integration. Renewable and
sustainable energy reviews.

[2] Elalfy D. A., Gouda E., Kotb M. F., Bureš V., Sedhom B. E. (2024). Comprehensive review of energy storage systems technologies,
objectives, challenges, and future trends. Energy
Strategy Reviews.

[3] Masias A., Marcicki J., Paxton W. A. (2021). Opportunities and
challenges of lithium
ion batteries in automotive applications. ACS
energy letters.

[4] Hasan M., Haque R., Jahirul M., Rasul M. G., Fattah I., Hassan N., Mofijur M. (2025). Advancing energy storage:
The future
trajectory of lithium-ion battery technologies. Journal of Energy Storage.

[5] Ngoy K. R., Lukong V. T., Yoro K. O., Makambo J. B., Chukwuati N. C., Ibegbulam C., Eterigho-Ikelegbe O., Ukoba K., Jen T.-C. (2025). Lithium-ion
batteries and the future of sustainable energy: A comprehensive review. Renewable and Sustainable Energy Reviews.

[6] Skyllas-Kazacos M., Rychcik M., Robins R. G., Fane A., Green M. (1986). New all-vanadium
redox flow cell. J. Electrochem. Soc..

[7] Zhang L., Yu G. (2023). Recent developments
in materials and chemistries for redox flow batteries. ACS Mater. Lett..

[8] Minke C., Kunz U., Turek T. (2017). Techno-economic
assessment of novel
vanadium redox flow batteries with large-area cells. J. Power Sources.

[9] Hu H., Han M., Liu J., Zheng K., Mu Y., Zou Z., Yu F., Li W., Zhao T. (2024). Development status, challenges, and
perspectives of key components and systems of all-vanadium redox flow
batteries. Future Batteries.

[10] Huang Z., Mu A., Wu L., Yang B., Qian Y., Wang J. (2022). Comprehensive
analysis of critical issues in all-vanadium redox flow battery. ACS sustainable chemistry & engineering.

[11] Ye L., Qi S., Cheng T., Jiang Y., Feng Z., Wang M., Liu Y., Dai L., Wang L., He Z. (2024). Vanadium redox flow
battery: review and perspective of 3D electrodes. ACS Nano.

[12] Zhao Y., Kang Y., Wozny J., Lu J., Du H., Li C., Li T., Kang F., Tavajohi N., Li B. (2023). Recycling
of sodium-ion batteries. Nature Reviews Materials.

[13] Wu H., Hao J., Jiang Y., Jiao Y., Liu J., Xu X., Davey K., Wang C., Qiao S.-Z. (2024). Alkaline-based aqueous
sodium-ion batteries for large-scale energy storage. Nat. Commun..

[14] Yao A., Benson S. M., Chueh W. C. (2025). Critically
assessing sodium-ion technology
roadmaps and scenarios for techno-economic competitiveness against
lithium-ion batteries. Nature Energy.

[15] Voß P., Gruber B., Mitterfellner M., Plöpst J.-D., Degen F., Schmuch R., Lux S. (2025). Benchmarking state-of-the-art
sodium-ion battery cells–modeling energy density and carbon
footprint at the gigafactory-scale. Energy Environ.
Sci..

[16] Lopes P. P., Stamenkovic V. R. (2020). Past, present,
and future of lead–acid batteries. Science.

[17] Zhang Y., Zhou C.-G., Yang J., Xue S.-C., Gao H.-l., Yan X.-H., Huo Q.-Y., Wang S.-W., Cao Y., Yan J. (2022). Advances and challenges in improvement of the electrochemical performance
for lead-acid batteries: a comprehensive review. J. Power Sources.

[18] Vangapally N., Penki T. R., Elias Y., Muduli S., Maddukuri S., Luski S., Aurbach D., Martha S. K. (2023). Lead-acid batteries
and lead–carbon hybrid systems: A review. J. Power Sources.

[19] Yan Z., Zhao L., Wang Y., Zhu Z., Chou S. L. (2022). The Future
for Room-Temperature Sodium–Sulfur Batteries: From Persisting
Issues to Promising Solutions and Practical Applications. Adv. Funct. Mater..

[20] Wang Y., Zhou D., Palomares V., Shanmukaraj D., Sun B., Tang X., Wang C., Armand M., Rojo T., Wang G. (2020). Revitalising sodium–sulfur batteries for non-high-temperature
operation: a crucial review. Energy Environ.
Sci..

[21] Zhao L., Tao Y., Zhang Y., Lei Y., Lai W. H., Chou S., Liu H. K., Dou S. X., Wang Y. X. (2024). A Critical Review
on Room-Temperature Sodium-Sulfur Batteries: From Research Advances
to Practical Perspectives. Adv. Mater..

[22] Lee D., Kim B., Kim D. (2025). Vanadium ion
battery (VIB) for grid-scale energy storage. J. Power Sources.

[23] Nasimifar A., Mehrabani J. V. (2022). A review
on the extraction of vanadium pentoxide from
primary, secondary, and co-product sources. Int. J. Mining Geo-Eng..

[24] An Y., Ma B., Li X., Chen Y., Wang C., Wang B., Gao M., Feng G. (2023). A review on the roasting-assisted leaching and recovery
of V from vanadium slag. Process Safety and
Environmental Protection.

[25] Moskalyk R., Alfantazi A. (2003). Processing of vanadium: a review. Miner. Eng..

[26] Liu S., Chen Y., Yu S., Zhang D., Xie G. (2022). Rapid Vanadium
Extraction from Roasted Vanadium Steel Slag via a H2SO4–H2O2
System: Process and Mechanism. ACS Omega.

[27] Gao F., Olayiwola A. U., Liu B., Wang S., Du H., Li J., Wang X., Chen D., Zhang Y. (2022). Review of vanadium
production part I: primary resources. Mine.
Process. Extract. Metallurg. Rev..

[28] Wen J., Jiang T., Xu Y., Liu J., Xue X. (2018). Efficient
separation and extraction of vanadium and chromium in high chromium
vanadium slag by selective two-stage roasting–leaching. Metallurg. Mater. Trans. B.

[29] Li H.-Y., Fang H.-X., Wang K., Zhou W., Yang Z., Yan X.-M., Ge W.-S., Li Q.-W., Xie B. (2015). Asynchronous
extraction of vanadium and chromium from vanadium slag by stepwise
sodium roasting–water leaching. Hydrometallurgy.

[30] Wang J., Zhang P., Wang S., Yang L., Luo J., Shen B. (2021). Mechanisms and kinetics
of a new cleaner single cyclic roasting-leaching
process for the extraction of vanadium from Linz–Donawitz converter
slag using CaCO3 and H2SO4. Clean. Eng. Technol..

[31] Wang Y.-H., Wang Y.-F., Li Y.-T., Wu C., Han X.-L., Zhao N.-N., Zhang Z.-K., Dai L., Wang L., He Z.-X. (2024). A review on vanadium extraction techniques
from major vanadium-containing
resources. Rare Met..

[32] Liu S., Wang L., Chen J., Ye L., Du J. (2024). Research progress
of vanadium extraction processes from vanadium slag: A review. Sep. Purif. Technol..

[33] Park J. H., Park J. J., Lee H. J., Min B. S., Yang J. H. (2018). Influence
of metal impurities or additives in the electrolyte of a vanadium
redox flow battery. J. Electrochem. Soc..

[34] Becker H., Murawski J., Shinde D. V., Stephens I. E., Hinds G., Smith G. (2023). Impact of impurities
on water electrolysis: a review. Sustainable
Energy & Fuels.

[35] Zhou H., Liu W., Hao D., Hong H., Wang Y., Zhu Q., Wang L., Jiang Y., Feng Z., He Z. (2024). Recent Advances
and Perspectives of Impurity Ions and Additives for the Electrolyte
of Vanadium Redox Flow Battery. Energy Fuels.

[36] Cao L., Skyllas-Kazacos M., Menictas C., Noack J. (2018). A review of electrolyte
additives and impurities in vanadium redox flow batteries. Journal of energy chemistry.

[37] Yuan X. Z., Song C., Platt A., Zhao N., Wang H., Li H., Fatih K., Jang D. (2019). A review of all-vanadium redox flow
battery durability: Degradation mechanisms and mitigation strategies. Int. J. Energy Res..

[38] Pichugov R., Loktionov P., Verakso D., Pustovalova A., Chikin D., Antipov A. (2024). Sensitivity of Capacity Fade in Vanadium
Redox Flow Battery to Electrolyte Impurity Content. ChemPlusChem..

[39] Pahlevaninezhad M., Pahlevani M., Roberts E. P. (2022). Effects of aluminum, iron, and manganese
sulfate impurities on the vanadium redox flow battery. J. Power Sources.

[40] Schweiss R., Pritzl A., Meiser C. (2016). Parasitic
hydrogen evolution at different
carbon fiber electrodes in vanadium redox flow batteries. J. Electrochem. Soc..

[41] Fan C., Yang H., Zhu Q. (2017). Preparation and electrochemical properties
of high purity mixed-acid electrolytes for high energy density vanadium
redox flow battery. Int. J. Electrochem. Sci..

[42] An Y., Ma B., Zhou Z., Chen Y., Wang C., Wang B., Gao M., Feng G. (2023). Extraction of vanadium from vanadium slag by sodium
roasting-ammonium sulfate leaching and removal of impurities from
weakly alkaline leach solution. Journal of Environmental
Chemical Engineering.

[43] Li C., An X., Jiang T., Wen J. (2025). Efficient recovery
of vanadium from
calcified vanadium slag by reductive leaching-selective precipitation:
Thermodynamic and kinetic analysis and mechanism investigation. Sep. Purif. Technol..

[44] Zhang L., Du J., Wang L. (2025). A green and cost-effective
approach for high-purity
VOCl_3_ preparation and its application in high-performance
vanadium redox flow batteries. Journal of Energy
Storage.

[45] Yang X., Zhang Y., Bao S. (2016). Preparation of high purity V_2_O_5_ from a typical low-grade refractory stone coal
using a pyro-hydrometallurgical process. Minerals.

[46] Wen J., Jiang T., Zhou W., Gao H., Xue X. (2019). A cleaner
and efficient process for extraction of vanadium from high chromium
vanadium slag: Leaching in (NH_4_) _2_SO_4_-H_2_SO_4_ synergistic system and NH_4_
^+^ recycle. Sep. Purif. Technol..

[47] Li L., Gao X., Wang X., Qi J., Zhao B., Wan H. (2021). A novel method
to prepare high-purity V_2_O_5_ from Na_3_VO_4_ solution. Journal of Materials
Research and Technology.

[48] Ning P., Lin X., Wang X., Cao H. (2016). High-efficient
extraction of vanadium
and its application in the utilization of the chromium-bearing vanadium
slag. Chemical Engineering Journal.

[49] Yuan B., He K., Wu P., Liu C., He J., Jiang W. (2025). Novel Clean
Process for High-Purity Vanadium Production via Photocatalytic Chromium
Removal. Ind. Eng. Chem. Res..

[50] Hong H.-J., Kim H. S., Suh Y. J. (2022). Recovery of High-Purity
V (IV) and
V (III) Compounds from V (V) Sources by Reduction–Precipitation. Ind. Eng. Chem. Res..

[51] Guo Y., Yang Y., Li W., Wen J., Liu B. (2023). Novel Process
to Prepare a Vanadium Electrolyte from a Calcification Roasting–Acid
Leaching Solution of Vanadium Slag. Ind. Eng.
Chem. Res..

[52] Kaur A., Jeong K. I., Kim S. S., Lim J. W. (2022). Optimization
of
thermal treatment of carbon felt electrode based on the mechanical
properties for high-efficiency vanadium redox flow batteries. Compos. Struct..

