# Supplementary material for: Defining Metal-Impurity Thresholds for Hydrogen Evolution in Sealed Vanadium Ion Batteries
Source: ACS Omega. 2026 Feb 9;11(7):11569–77. doi: 10.1021/acsomega.5c09587 (PMC12947172; doi:10.1021/acsomega.5c09587)
Supplement: Supplementary file 1 [file ao5c09587_si_001.pdf]

Supporting Information

Defining Metal-Impurity Thresholds for Hydrogen Evolution in Sealed Vanadium Ion Batteries

Dongyoung Lee[1*], Bugi Kim[1], Eunhag Lee[1], Inwoo Cho[1], Dongheun Kim[1]

[1] Standard Energy, Daejeon, 34014, Republic of Korea.

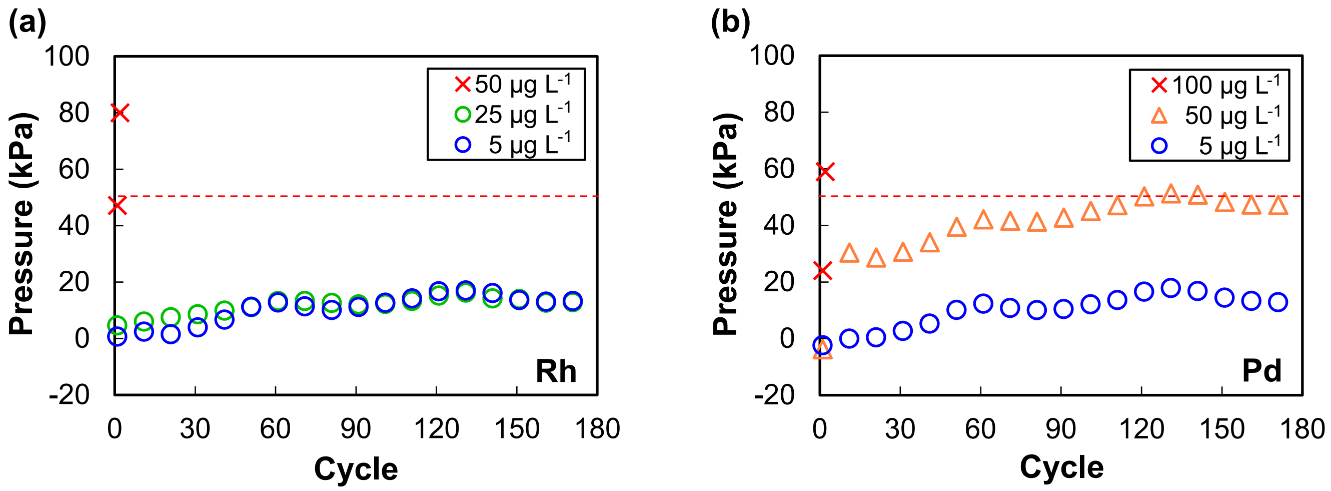

Figure S1. Class 1 impurity effects (a) Rhodium and (b) Palladium.

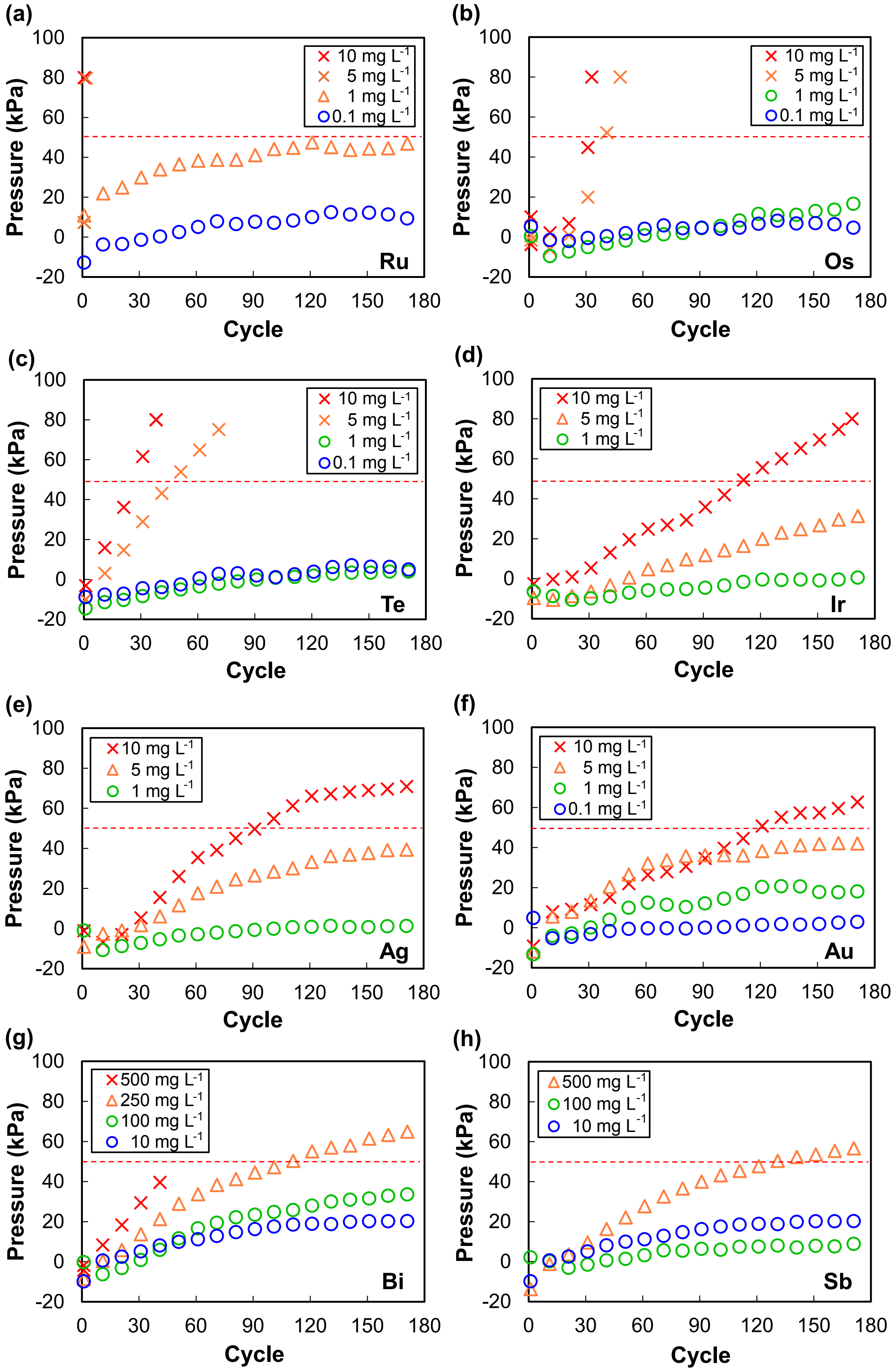

Figure S2. Class 2 impurity effects (a) Ruthenium, (b) Osmium, (c) Tellurium, (d) Iridium, (e) Silver, (f) Gold, (g) Bismuth, and (h) Antimony.

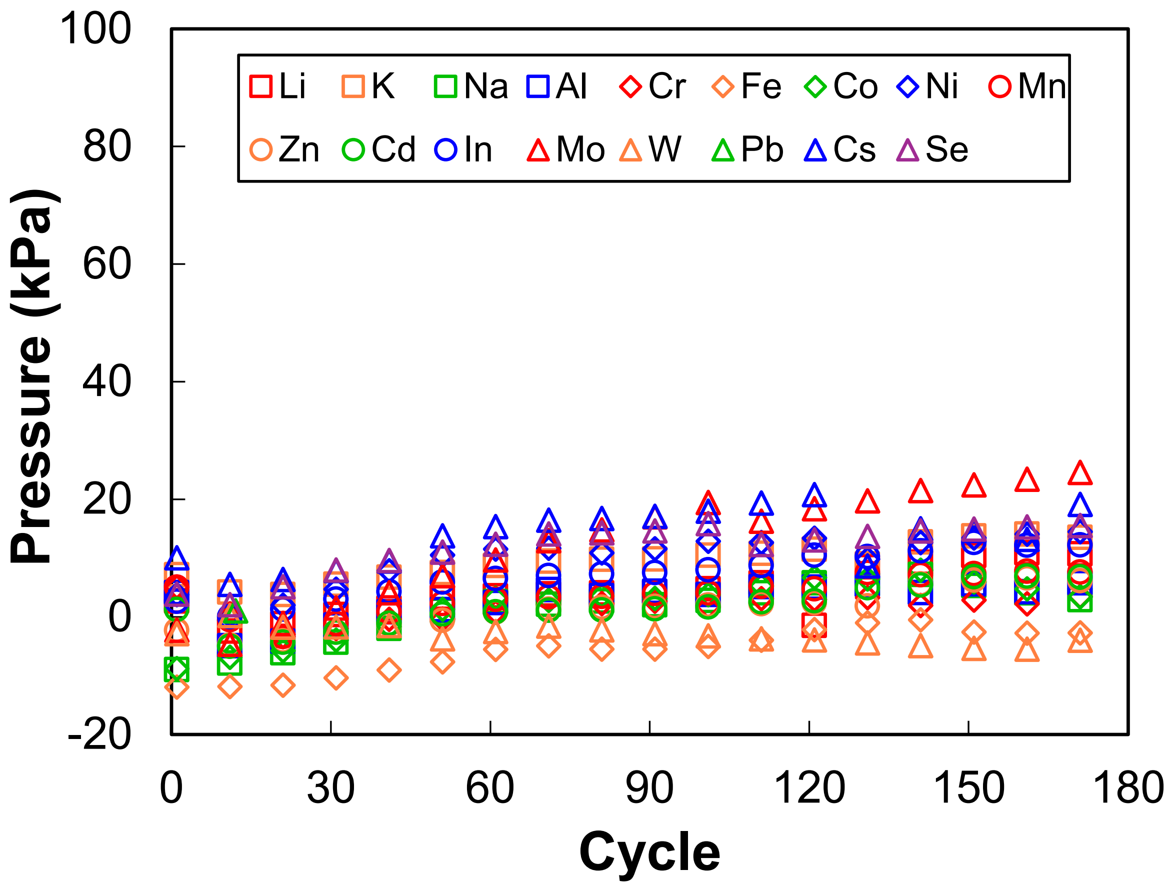

Figure S3. Class 3 and Class 4 impurity effects.

Table S1 Impurity ions and corresponding reagents used

Impurity Ion	Reagent	Formula	Purity	CAS No.
Pt^{4+}	Pt standard solution	Pt (in HCl)	99.9%	7440-06-4
Rh^{3+}	Rh standard solution	Rh (in HCl)	99.9%	7440-16-6
Pd^{2+}	Pd standard solution	Pd (in HCl)	99.9%	7440-05-3
Ru^{3+}/Ru^{4+}	Ru standard solution	Ru (in HCl)	99.9%	7440-18-8
Cu^{2+}	Copper(II) sulfate pentahydrate	$CuSO_4{\cdot}5H_2O$	99.0%	7758-99-8
Os^{4+}	Os standard solution	Os (in HCl)	99.9%	7440-04-2
Te^{4+}	Tellurium oxide	TeO_2	99.0%	7446-07-3
Ir^{3+}/Ir^{4+}	Ir standard solution	Ir (in HCl)	99.9%	7439-88-5
Ag^{+}	Silver sulfate	Ag_2SO_4	99.99%	10294-26-5
Au^{3+}	Au standard solution	Au (in HCl)	99.99%	7440-57-5
Bi^{3+}	Bismuth nitrate pentahydrate	$Bi(NO_3)_3{\cdot}5H_2O$	98.0%	10035-06-0
Sb^{3+}	Antimony(III) chloride	$SbCl_3$	98.5%	10025-91-9
Li^{+}	Lithium sulfate monohydrate	$Li_2SO_4{\cdot}H_2O$	98.5%	10102-25-7
K^{+}	Potassium phosphate	K_3PO_4	99.0%	7778-53-2
Na^{+}	Sodium sulfate	Na_2SO_4	99.0%	7757-82-6
Al^{3+}	Aluminum sulfate octadecahydrate	$Al_2(SO_4)_3{\cdot}18H_2O$	97.0%	7784-31-8
Cr^{3+}	Chromium(III) oxide	Cr_2O_3	99.0%	1308-38-9
Fe^{2+}	Iron(II) sulfate heptahydrate	$FeSO_4{\cdot}7H_2O$	98.0%	7782-63-0
Co^{2+}	Cobalt(II) sulfate heptahydrate	$CoSO_4{\cdot}7H_2O$	98.0%	10026-24-1
Ni^{2+}	Nickel powder	Ni	99.9%	7440-02-0
Mn^{2+}	Manganese sulfate pentahydrate	$MnSO_4{\cdot}5H_2O$	98.0%	15244-36-7
Zn^{2+}	Zinc sulfate heptahydrate	$ZnSO_4{\cdot}7H_2O$	99.0%	7446-20-0
Cd^{2+}	Cadmium(II) chloride monohydrate	$CdCl_2{\cdot}H_2O$	95.0%	35658-65-2
In^{3+}	Indium(III) sulfate nonahydrate	$In_2(SO_4)_3{\cdot}9H_2O$	99.99%	13464-82-9
Mo^{6+}	Sodium molybdate dihydrate	$Na_2MoO_4{\cdot}2H_2O$	98.5%	10102-40-6
W^{6+}	Sodium tungstate dihydrate	$Na_2WO_4{\cdot}2H_2O$	98.0%	10213-10-2
Pb^{2+}	Lead(II) chloride	$PbCl_2$	98.0%	7758-95-4
Si^{4+}	Sodium metasilicate pentahydrate	$Na_2SiO_3{\cdot}5H_2O$	99.0%	10213-79-3
Cs^{+}	Cesium sulfate	Cs_2SO_4	99.0%	10294-54-9
Se^{4+}	Selenious acid	H_2SeO_3	97.0%	7783-00-8

Table S2 Impurity level for reference liquid electrode analyzed by ICP-OES

No.	Element	ppm	mg L^{-1}
1	Fe	62.1	88.1
2	Na	59.3	84.0
3	Al	25.3	35.8
4	Cr	7.9	11.1
5	K	3.6	5.2
6	Zn	1.7	2.5
7	Ag	N.D	N.D
8	Au	N.D	N.D
9	Bi	N.D	N.D
10	Cd	N.D	N.D
11	Co	N.D	N.D
12	Cs	N.D	N.D
13	Cu	N.D	N.D
14	In	N.D	N.D
15	Ir	N.D	N.D
16	Li	N.D	N.D
17	Mn	N.D	N.D
18	Mo	N.D	N.D
19	Ni	N.D	N.D
20	Os	N.D	N.D
21	Pb	N.D	N.D
22	Pd	N.D	N.D
23	Pt	N.D	N.D
24	Rh	N.D	N.D
25	Ru	N.D	N.D
26	Sb	N.D	N.D
27	Se	N.D	N.D
28	Si	N.D	N.D
29	Te	N.D	N.D
30	W	N.D	N.D

N.D - Not Detected
